# VISION IMPAIRMENT AND COGNITION IN INDIA: ASSOCIATIONS AFTER ADJUSTMENT FOR POTENTIAL BIAS

**DOI:** 10.1093/geroni/igad104.3711

**Published:** 2023-12-21

**Authors:** Emma Nichols, Yizhou Chen, Adina Zeki Al Hazzouri, Alden Gross, Niranjani Nagarajan, Jinkook Lee, Joshua Ehrlich

**Affiliations:** University of Southern California, Los Angeles, California, United States; University of Southern California, Los Angeles, California, United States; Columbia University, New York City, New York, United States; Johns Hopkins University Bloomberg School of Public Health, Baltimore, Maryland, United States; Michigan Medicine, Ann Arbor, Michigan, United States; University of Southern California, Los Angeles, California, United States; University or Michigan, Ann Arbor, Michigan, United States

## Abstract

Increasing evidence suggests that vision impairment may be an important modifiable risk factor for dementia, particularly in low- and middle-income settings where the prevalence of uncorrected vision impairment is high. Although prior studies in various settings, including India, have found strong associations between vision impairment and cognition, this work has not considered potential bias in cognitive testing due to vision impairment. We evaluated and adjusted for measurement differences by vision impairment status using data from the Longitudinal Aging Study in India–Diagnostic Assessment of Dementia (LASI-DAD) study (N=3780). We used Multiple Indicators Multiple Causes models to assess differential item functioning (DIF) (e.g. bias) in cognitive testing by objective near and distance vision impairment. We estimated associations between vision impairment and cognition adjusting for hypothesized confounders before and after DIF adjustment. Although there was statistical evidence of DIF (near vision: 3/10 items, distance vision: 4/10 items), differences between DIF-unadjusted and -adjusted scores were small compared to the standard error of measurement, indicating no evidence of clinically meaningful measurement differences. Both near and distance vision impairment were associated with cognition before and after DIF-adjustment; after DIF-adjustment, severe near and distance vision impairment were associated with -0.43 [95% CI -0.53--0.33] and -0.60 [-0.76--0.43] standard deviation units lower cognitive scores compared to those with normal vision, respectively. In well-conducted large-scale surveys, bias in cognitive testing due to vision impairment is likely minimal, even in low- and middle-income settings. Findings strengthen the evidence base on vision impairment as a risk factor for dementia.

